# Anaplastic Lymphoma Kinase (ALK) Receptor Tyrosine Kinase: A Catalytic Receptor with Many Faces

**DOI:** 10.3390/ijms19113448

**Published:** 2018-11-02

**Authors:** Hao Huang

**Affiliations:** 1 Department of Pediatric Oncology, Dana-Farber Cancer Institute, Boston, MA 02215, USA; hao_huang@dfci.harvard.edu or huanghaoacademic@gmail.com; Tel.: +1-617-582-8046; 2 Department of Pediatrics, Harvard Medical School, Boston, MA 02115, USA

**Keywords:** ALK, ALK kinase inhibitors, cancers, aberrant forms, ALK fusion proteins, neuroblastoma, targeted therapy

## Abstract

The anaplastic lymphoma kinase (ALK) receptor is a membrane-bound tyrosine kinase. The pathogenesis of several cancers is closely related to aberrant forms of ALK or aberrant ALK expression, including ALK fusion proteins, ALK-activated point mutations, and ALK amplification. Clinical applications of different ALK inhibitors represent significant progress in targeted therapy. Knowledge of different aspects of ALK biology can provide significant information to further the understanding of this receptor tyrosine kinase. In this mini-review, we briefly summarize different features of ALK. We also summarize some recent research advances on ALK fusion proteins in cancers.

## 1. Introduction

In 1994, anaplastic lymphoma kinase (ALK) was first found as a tyrosine kinase in anaplastic large-cell lymphoma (ALCL) cell lines [1,2]. In these cell lines, ALK fusion proteins (NPM–ALK) resulting from chromosomal translocation were found [1,2]. After the discovery of the first ALK fusion protein, researchers started to investigate the receptor tyrosine kinase itself. In 1997, several studies reported essential findings related to wild-type ALK [3,4].

The human *ALK* gene is located at chromosome region 2p23.2–p23.1. This gene, which contains 26 exons, encodes the full-length ALK protein with 1620 amino acids. ALK is an enzyme with tyrosine kinase activity, which catalyzes the transference of a gamma-phosphate group from adenosine triphosphate (ATP) to a tyrosine residue on a substrate protein. Therefore, it catalyzes a tyrosine residue phosphorylation reaction on its substrate proteins. The phosphorylation and dephosphorylation of proteins are critical reactions catalyzed by different enzymes (kinases and phosphatases), which play critical roles in various cellular functions.

**Figure 1:**
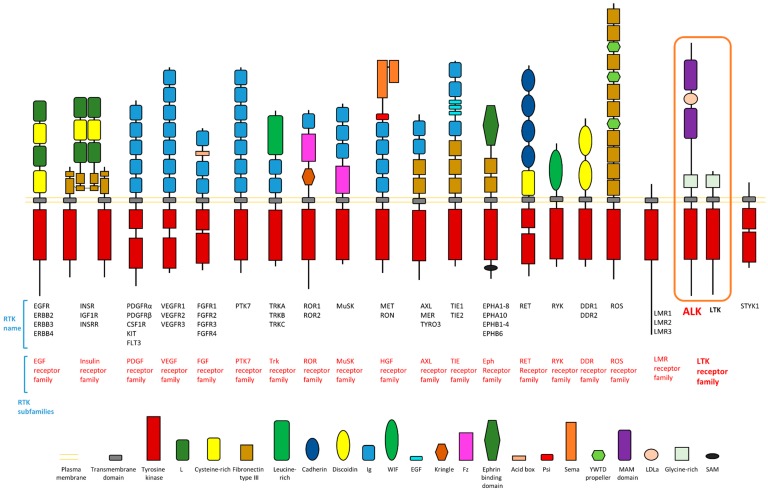
Domain structure of receptor tyrosine kinase families with anaplastic lymphoma kinase (ALK) highlighted. Modified from reference [5] with permission from Elsevier.

As one member of the receptor tyrosine kinase (RTK) family, ALK contains an extracellular domain (ECD), a transmembrane domain, and an intracellular domain (ICD) (Figure 1). There are more than 50 RTKs encoded in the human genome. These RTKs are grouped into 20 RTK subfamilies within the RTK family (Figure 1) [5]. All RTKs contain an extracellular region, a transmembrane domain, and intracellular domain (Figure 1). The tyrosine kinase domain of RTKs exists in the ICD (Figure 1). The ECD of RTKs usually varies in composition between the different RTK subfamilies (Figure 1). ALK belongs to the leukocyte tyrosine kinase (LTK) receptor subfamily (Figure 1), which includes two members: LTK and ALK. Based on the information on homology, the receptor LTK has the most similar features to ALK, although they differ in domain structure (Figure 1) [4,5]. Figure 1 shows the domain structure of human ALK and RTKs. ALK is a unique RTK member among the RTKs because the ALK ECD contains an extracellular domain structure, which does not exist in any other RTK member, including LTK (Figure 1). Detailed information is introduced in a subsequent section. RTKs are considered a large group of proteins called catalytic receptors, or enzyme-linked receptors [6]. Catalytic receptors are a large group of cell-surface proteins which bind to their ligands as cell-surface receptors in addition to carrying out their catalytic function [6]. Their roles, as both receptors and enzymes, are usually essential for the biological functions of RTKs. Numerous RTKs play an important role in transmembrane signaling and intercellular communication.

ALK is usually expressed during the development of the nervous system [4,7]. During mouse development, ALK expression was found in the central and peripheral nervous system, such as spinal cord motoneurons, sympathetic ganglia, and dorsal root ganglia [3,7]. A recent study showed that ALK was expressed by sympathetic neuroblasts during some stages (E12.5 and E13.5 stage) of mouse embryonic development [8]. After the birth of the mouse, the ALK expression level in the nervous system decreased. Additionally, during the development of chicks, ALK expression was found in the developing central and peripheral nervous system, including spinal cord motoneurons, sympathetic ganglia, and dorsal root ganglia [9]. In adult mammals, a relatively low level of ALK expression exists in certain regions of a few organs, such as the hippocampus within the brain [4,7,10,11]. Studies have shown that ALK is expressed in several regions of the hippocampus in the mouse brain, including the dentate gyrus, cornu ammonis (CA) 1 region, and CA3 region [10].

Although it is highly possible that the biological functions of mammalian ALK are related to the development and function of the nervous system, the direct biological roles of ALK are still not completely clarified. The study of *Alk* gene knockout mice indicates that ALK can affect the mouse brain functions [11,12,13,14]. Some behaviors closely related to brain functions were observed to differ between *Alk* gene knockout mice and wild-type mice [11,12,13,14]. For instance, several studies showed that *Alk* knockout mice displayed elevated ethanol consumption compared to wild-type mice [12,14].

**Figure 2:**
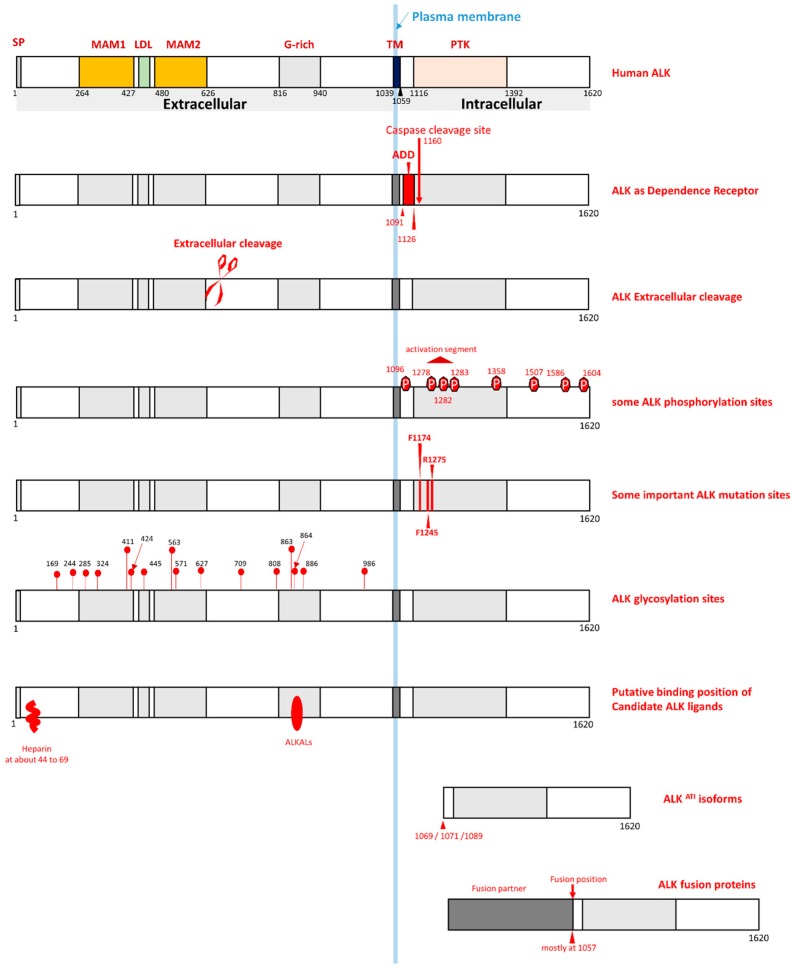
Summary of several ALK features. SP: Signal peptide; TM: Transmembrane domain; PTK: Protein kinase domain; G-rich: Glycine-rich domain; MAM: MAM domain; LDL: LDLα domain; ADD: Addiction/dependence domain.

This mini-review presents information on different aspects of ALK. Because several features of ALK biology are summarized and described in this review, a summarized illustration of these ALK features is presented (Figure 2).

## 2. ALK Domain Structure and 3-D Structure

Although ALK possesses characteristics that are common among RTKs, it also contains some unique features in its domain structure. The ECD of ALK is composed of 1038 amino acid residues (amino acids 1–1038) and has unique features (Figure 1 and Figure 2). In the ALK ECD, a low-density lipoprotein receptor class A domain (LDL, amino acids 453–471) is surrounded by two MAM domains (meprin/A5-protein/PTPmu; amino acids 264–427, 480–626) (Figure 2). In addition, the ALK ECD contains an N-terminal signal peptide (amino acids 1–18) and a glycine-rich region (amino acids 816–940) (Figure 2). ALK has a single transmembrane domain (amino acids 1039–1059). The ICD (amino acids 1060–1620) of ALK mainly comprises a tyrosine kinase domain (amino acids 1116–1392) and the juxtamembrane region (amino acids 1060–1115). In fact, ALK is the only RTK member that contains two MAM domains within its ECD. Among the RTKs, the combination of two MAM domains and one LDL domain is also unique to ALK. The biological roles of the LDL domain and the MAM domain are not yet clarified. The MAM domain consists of about 170 amino acid residues. Studies of the MAM domain in other cell surface proteins have shown that MAM domains usually participate in cell–cell interactions through homophilic binding [15,16,17]. The glycine-rich region of ALK contains consecutive glycine residues, but the function of the glycine-rich region within human ALK is still not clear. Previous studies using a *Drosophila melanogaster* model have shown that point mutations of glycine residues within the glycine-rich region of *Drosophila* Alk can lead to a loss of function [18].

**Figure 3:**
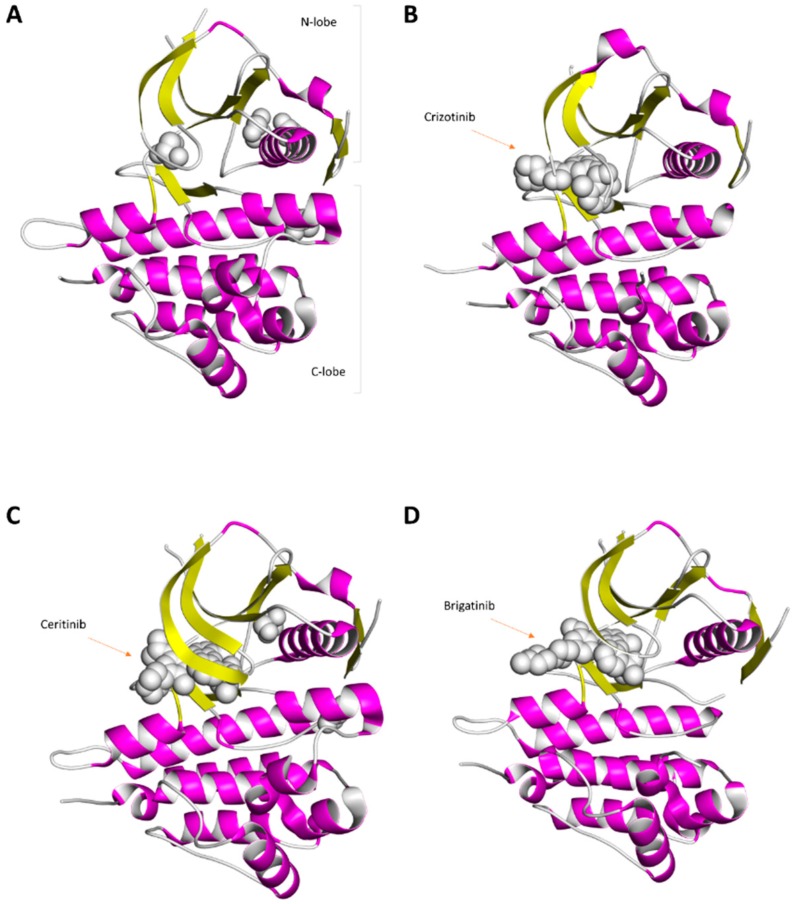
Ribbon diagram of the human ALK catalytic domain structure with or without ALK inhibitors. (**A**) Crystal structures of the ALK catalytic domain (PDB ID: 3L9P). This structure contains glycerol molecules (gray small molecules). (**B**) Ribbon diagram depicting the crystal structure of the ALK catalytic domain in complex with crizotinib (PDB ID: 2XP2). (**C**) Ribbon diagram depicting the crystal structure of the ALK catalytic domain in complex with ceritinib (PDB ID: 4MKC). (**D**) Crystal structure of the ALK catalytic domain bound to brigatinib (PDB ID: 5J7H).

The complete structure of ALK is still not known, nor is the structure of the entire ALK ECD. Most structural research has focused on the ALK kinase domain, which directly conducts enzyme catalysis [19,20,21,22,23,24]. This kinase domain contains an amino-terminal lobe and a carboxy-terminal lobe [19,20,21,22,23,24] (Figure 3). The amino-terminal lobe of the ALK kinase domain contains several β-strands (forming antiparallel β-sheets), several loop regions (including a glycine-rich loop), and one helix (αC-helix) [19,20,21,22,23,24] (Figure 3). The carboxy-terminal lobe contains several α-helices, two short β-strands, and several loop regions (Figure 3). In this kinase domain, the catalytic activity of ALK in the kinase structure is affected by several essential segments, which include a catalytic loop, activation loop, αC-helix, glycine-rich loop, etc. [19,20,21,22,23,24]. The crucial residues in the human ALK kinase domain include E1167 within the αC-helix, HRD residues (H1247, R1248, D1249) within the catalytic loop, K1150 within the N-lobe, DFG residues (D1270, F1271, G1272) within the activation segment, the K1267 residue, etc. [19,20,21,22,23,24]. In Figure 3, the structure of the ALK kinase domain is displayed. Structures of the ALK kinase domain bound with three ALK inhibitors are also shown in this figure (Figure 3).

## 3. ALK Activation and Signaling Pathway

One property of an RTK is its mediation of downstream signaling pathways after it has been activated. The exact activation mechanism of ALK is still not completely understood, but may be achieved through the canonical RTK activation mechanism. The canonical model of RTK activation is through ligand-induced activation. After the extracellular region of one RTK binds to its ligand present in the extracellular space, homo-dimerization or hetero-dimerization induced by ligand binding occurs. This dimerization results in the trans-phosphorylation of specific tyrosine residues within the cytoplasmic domain of the RTK, which may lead to more tyrosine residues being phosphorylated on the same RTK. This phosphorylation can then activate the catalytic capability of the RTK. Activated RTK can then phosphorylate tyrosine residues on its substrate proteins, which can transmit the signals of the RTK. Several mechanisms can terminate an activated RTK once it has been activated, including dephosphorylation by tyrosine phosphatases and degradation after endocytosis. Dimerization of ALK may support the trans-phosphorylation of some tyrosine residues (probable sites are Y1278, Y1282, and Y1283) in the activation loop [24,25,26,27,28]. Then, other tyrosine residues can be phosphorylated after dimerization to activate ALK kinase activity [24,25,26,27,28]. Like other protein kinases, activated ALK can activate downstream pathways. The NPM–ALK fusion proteins were found to activate several downstream signal pathways. These pathways include the RAS/MAPK pathway, the JAK/STAT pathway, the PI3K/Akt pathway, and the PLC (phospholipase C)-γ pathway [24,25,26,27,28]. Activation of these pathways by NPM–ALK is completed through the phosphorylation of specific tyrosine residues of ALK. These residues include, but are not limited to, the tyrosine residues corresponding to Y1358, Y1507, and Y1604 of the full-length ALK (Figure 2). Phosphorylation of these ALK residues can transmit ALK-mediated signals to downstream signaling pathways [24,25,26,27,28].

**Figure 4:**
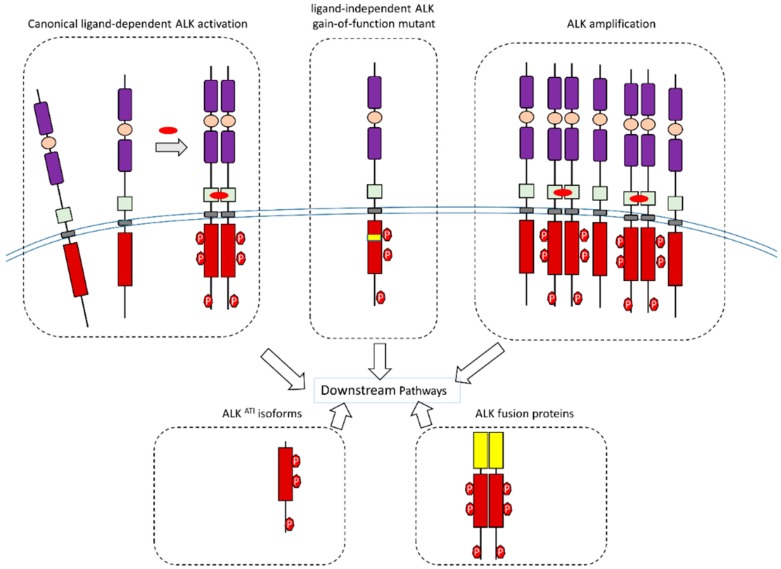
Schematic illustration of wild-type ALK, aberrant forms of ALK, ALK amplification, and ALK isoforms during signal transduction.

In addition to canonical wild-type ALK activation, aberrant forms of ALK and ALK isoforms also can transmit signals to their downstream pathways (Figure 4).

## 4. ALK Ligands

To date, several potential human ALK ligands have been discovered (Figure 2), but more research is required to clarify all information of human ALK ligands. Recent research has discovered that ALKALs are ALK ligands [29,30,31,32,33,34]. These studies have shown that ALKALs (FAM150A and FAM150B), which were also found to be ligands of LTK, bind to the ALK ECD to activate ALK [29,30,31,32,33,34]. In vitro studies have shown that ALKALs activate ALK kinase activity. Conditioned medium containing ALKALs activated ALK in several ALK-expressing cell lines [30,31]. Expression of ALKALs also led to the activation of wild-type ALK in a *Drosophila* model. Additionally, in vivo studies using zebrafish as a model also supported ALKALs as ligands of the ALK/LTK receptor family [32,33]. Heparin was also found to be a putative ligand of mammalian ALK in a study [35]. A putative heparin-binding motif was found in the N-terminal region of the ALK ECD. Additionally, an in vitro study using canine ALK showed that the wild-type ALK ECD, but not a mutant ALK ECD with its N-terminal region deleted, could be purified using heparin–Sepharose chromatography. Heparins with a relatively long chain, such as heparins whose chains have a degree of polymerization (DP) of 25, can physically bind to the ALK ECD and activate ALK [35].

In *Drosophila melanogaster*, jelly belly (Jeb) has already been discovered as a biological ligand of Alk, which can activate it to promote visceral founder cell specification during the visceral musculature development of the gut [18,36,37]. During fruit fly embryogenesis, dAlk appears to function in gut development in *Drosophila* by activating its downstream signaling, such as the ERK signaling pathway [18,36,37]. Without a functional *Alk* gene in the fruit fly, the development of the gut is disrupted [18,36,37]. In addition, dAlk and its ligand Jeb play a critical role in the development of the visual system [38].

## 5. ALK Is a Dependence Receptor

One crucial characteristic of ALK is that it is a so-called dependence receptor [39,40,41] (Figure 2). Without ligand binding to activate its kinase activity, ALK can be cleaved by caspase-3 during apoptosis [39,40,41]. In the juxtamembrane region of ALK, there is a caspase-3 cleavage site (amino acids 1160–1163: DELD) (Figure 2). Elevated caspase-3 activity can cleave this ALK at this cleavage site, which releases an intracellular ALK fragment (about 60 kDa) into the cytoplasm. This caspase-dependent cleavage of ALK enhances apoptosis through the exposure of a pro-apoptotic segment (addiction/dependence domain, ADD) within the ALK juxtamembrane region [39,40,41]. ALK mutant D1160N abolishes the caspase-3 cleavage at this cleavage position, which also abrogates the ALK-mediated enhancement of apoptosis [39,40,41]. Moreover, people have found that synthesized peptides mimicking the proapoptotic domain of ALK caused cytotoxicity to ALK-positive ALCL and neuroblastoma (NB) cell lines [41]. This cytotoxic effect was found to be due to caspase-dependent apoptosis [41]. ALK is not the only RTK member belonging to the dependence receptor group [42,43,44,45,46]. Several other RTKs, such as MET, RET, and TrkC, are also dependence receptors that can be cleaved by caspases to enhance apoptosis [42,43,44,45,46,47]. Because this is a significant characteristic of ALK, studies on this topic could be a critical ALK research area.

## 6. ALK Extracellular and Intracellular Cleavage

Human ALK exists as a 220 kDa full-length ALK and a 140 kDa truncated ALK [48]. The full-length wild-type ALK can be cleaved in the ALK ECD (Figure 2). The extracellular cleavage of ALK results in a 140 kDa truncated ALK and a fragment shed into the extracellular space. The ALK ECD cleavage phenomenon can be detected in the developing brain of rats in vivo [49]. Also, this cleavage phenomenon has been frequently found in NB cancer cell lines and NB cancer tissues [48,49,50,51,52]. Previous research indicates that, during the development of rat dorsal root ganglion (DRG), ALK cleavage is regulated by Schwann cells [49].

The ALK ICD can also be cleaved by caspase-3 [39,40,41] (Figure 2). During apoptosis, active caspase-3 can cleave ligand-free ALK at a caspase cleavage site located in the juxtamembrane region [39,40,41].

## 7. ALK Glycosylation

One critical post-translational modification of ALK is *N*-glycosylation (Figure 2). There are 16 *N*-glycosylation sites within the ECD of ALK (Figure 2). As a result of ALK glycosylation, the molecular weight of the full-length wild-type ALK dramatically increases. The full-length ALK is about 180 kDa without glycosylation. After glycosylation, the molecular weight of the full-length ALK displayed on an SDS-PAGE gel is about 220 kDa. With respect to membrane glycoproteins, *N*-glycosylation typically contributes to glycoprotein folding, protein quality control, and membrane trafficking [53,54,55,56]. *N*-glycosylation of ALK may be involved in its function in protein folding, protein quality control, and membrane anchoring. One study indicated that inhibition of *N*-glycosylation of ALK negatively affected ALK phosphorylation and its downstream signaling [57].

## 8. ALK Isoforms

A recent study discovered an alternative transcription initiation (ATI) site in intron 19 of the *ALK* gene [58]. This ATI transcript results in the existence of three ALK isoforms (ALK^ATI^) with molecular weights of 61.1, 60.8, and 58.7 kDa [58] (Figure 2). These three isoforms may result from the existence of three predicted in-frame translation start codons (ATGs) in the ALK^ATI^ transcript [58].

ALK^ATI^ isoforms are expressed in about 2% to 3% of melanomas. It is also sporadically expressed in several other human cancers, such as lung adenocarcinoma and kidney renal clear cell carcinoma [58]. The ALK^ATI^ isoforms, which are kinase active, contain oncogenic capacity [58]. An in vitro study showed that ALK^ATI^ could drive growth-factor-independent cell proliferation. Also, research using a mouse model showed that ALK^ATI^ promoted tumorigenesis [58]. The ALK inhibitor crizotinib efficiently inhibited both ALK^ATI^ kinase activity and ALK^ATI^ tumorigenesis ability [58]. An in vitro study using Ba/F3 cells stably expressing ALK isoforms, showed that ALK inhibitors ceritinib and TAE-684 also inhibited the IL-3-independent growth of the transformed Ba/F3 cells.

The positions of three translational start codons (ATG 1069, 1071, and 1089) in ALK alternative transcription initiation are not far from the codon that encodes D1160. Therefore, the molecular weights of these ALK isoforms are a little higher than that of the ALK fragment that is released into the cytoplasm after caspase-3 cleavage. ALK^ATI^ isoforms exist in the cytoplasm and the nucleus. Within the chromatin ATI region of ALK, there are two transposable elements, which include a long-terminal repeat (LTR) and a long interspersed nuclear element (LINE).

The existence of ALK isoforms indicates that ALK may not only function as a membrane binding RTK but also as a cytoplasmic tyrosine kinase when these isoforms of ALK are expressed. It is still not clear what the biological roles of these ALK isoforms are, nor the mechanisms which determine this alternative transcription.

## 9. Aberrant Forms of ALK and Aberrant ALK Expression in Cancers

Aberrant forms of ALK have been found in various cancers. Aberrant forms of ALK and aberrant ALK expression are generally caused by at least one of three primary mechanisms: ALK fusion mutations, ALK gain-of-function mutations, or ALK amplification.

ALK fusion proteins have been found be critical oncogenic drivers in some cancers, such as non-small cell lung cancer (NSCLC) [59,60], ALCL [1,2], and inflammatory myofibroblastic tumor (IMT) [61,62]. ALK fusion variants are usually caused by chromosomal translocation, which can lead to the creation of fusion proteins consisting of an ALK fragment and a fusion partner, such as NPM–ALK in ALCL and EML4–ALK in NSCLC [1,2,59,60]. Even in the same type of cancer, different ALK fusion proteins have been discovered. For instance, there are at least nine different ALK fusion proteins identified in ALCL [24,28]. Additionally, in some types of cancers, such as ALCL, the frequency of ALK rearrangements in patients is high [1,2,24,28,63,64]. ALK fusion proteins exist in more than 50% of ALCL cases [1,2,24,28,63,64]. NPM–ALK is the most frequently detected form of ALK fusion proteins in ALCL [1,2,24,28,63,64]. ALK fusion proteins are usually found to activate downstream signaling pathways that contribute to related cancer pathogenesis. Moreover, an increasing number of novel ALK fusion proteins are being identified in various types of cancers.

ALK activation mutation and ALK amplification were reported in pediatric cancer neuroblastoma many years ago [51,65,66,67,68]. Multiple ALK activation mutations were found in this cancer, which include, but are not limited to: ALK F1174I, ALK F1174L, ALK F1245C, ALK F1245V, ALK R1275Q, ALK R1275L, ALK D1091N, ALK G1128A, ALK M1166R, ALK I1171N, ALK R1192P, and ALK I1250T [51,65,66,67,68]. There are three major ALK mutated positions within the kinase domain: R1275, F1174, and F1245 [51,65,66,67,68]. These are three hotspot residues for ALK-activating point mutations. Both germline and somatic activating mutations have been found in neuroblastoma [51,65,66,67,68,69,70,71]. In addition to ALK-activated point mutations, truncated activated ALK mutants, including ALK Δ2–3, ALK Δ1–5, and ALK Δ4–11, have been found in several neuroblastoma-derived cell lines and tumor samples [72,73,74]. Additionally, one novel truncated form of an ALK variant (ALK Δ2–17) was identified recently in a ALK-positive anaplastic large cell lymphoma and one synovial sarcoma cell line [75,76]. The lack of several exons in *ALK* genes caused by genomic rearrangements leads to the generation of truncated ALK mutants [72,73,74]. Aberrant activation of ALK activity plays a crucial oncogenic role in neuroblastoma [51,65,66,67,68,77,78,79,80]. For instance, studies in multiple models have shown that aberrantly-activated ALK can potentiate the effect of another protein, MYCN, to drive neuroblastoma pathogenesis [52,77,78,79,80]. In addition to neuroblastoma, ALK amplification and ALK copy number gain have been found in other cancers, such as rhabdomyosarcomas [81,82]. Because several previous reviews also provide excellent summaries of aberrant ALK forms in cancers, this review does not present additional detailed information on this topic [24,28,64,71,83].

### ALK Fusion Proteins in Cancers

In ALK fusion proteins, the ALK fusion partner may cause dimerization (or oligomerization) of the ALK fusion protein independent of ligand binding, causing oncogenic ALK activation. This is one canonical mechanism that can explain why ALK fusion proteins cause ALK activation. Moreover, the ALK fusion partner may also affect the subcellular location of the ALK fusion proteins. Because ALK fusion partners provide the N-terminal region of the fusion proteins, the transcription of a fusion protein is usually regulated by the promoter of ALK’s partner protein. The breakpoints for the translocations of *ALK* genes are typically located at exons 19–20 or exons 20–21. ALK fusion proteins usually contain the complete ALK kinase domain. The kinase activity of ALK fusion proteins leads to the activation of downstream signaling pathways, such as the RAS/MAPK pathway and the JAK/STAT pathway.

Some excellent reviews already summarize the ALK fusion proteins found in various types of cancers [24,28,64,83]. Because an increasing number of ALK fusion proteins are being identified in different types of cancers, we summarize the novel ALK fusion proteins that were found recently (from 2016 to 2018) (Table 1). The existence of numerous diverse ALK fusion proteins suggests that the establishment of ALK fusion proteins through translocation is an important molecular mechanism of oncogenesis in multiple cancer types.

## 10. ALK Tyrosine Kinase Inhibitors

Because oncogenic activation of ALK kinase activity is crucial to ALK fusion proteins and ALK gain-of-function point mutants, inhibition of ALK kinase activity is the key to targeting ALK in various cancers. To date, multiple generations of ALK tyrosine kinase inhibitors (TKIs) have been generated and evaluated, most of which are small molecular inhibitors.

Inhibition of ALK kinase activity using ALK–TKIs has been found to have potent antitumor efficacy in various research [105,106,107,108,109,110,111,112]. Furthermore, researchers have generated several highly potent selective ALK–TKIs, which can inhibit multiple aberrant forms of ALK, including ALK fusion proteins and ALK activated mutants [108,112,113,114,115,116,117,118,119,120,121,122,123,124,125]. Several ALK–TKIs have already been approved for use in the clinical treatment of specific cancers in some countries. ALK inhibitors that were approved by the U.S. Food and Drug Administration (FDA) for specific cancers include ceritinib, crizotinib, alectinib, and brigatinib. Some information related to these ALK inhibitors is listed in Table 2.

For ALK-directed therapy using ALK-TKIs, acquired drug resistance always arises in some patients, even though these patients may initially experience partial response or complete response. Drug resistance mechanisms after a specific ALK-TKI treatment are investigated widely. One mechanism of resistance is caused by acquired secondary point mutations in the ALK kinase domain. This type of ALK mutations found in previous studies include, but are not limited to: ALK G1202R, ALK F1174 L, ALK F1174C, ALK L1196 M, ALK I1171T, ALK G1269S, ALK V1180L, and ALK G1269A [108,112,113,114,115,116,117,118,119,120,121,122,123,124,125]. Other mechanisms include the activation of alternative survival pathways, *ALK* gene amplification, etc. In specific situations, resistance mutations may emerge during ALK–TKI treatment, or resistance mutations may already exist before ALK–TKI treatment. Overcoming acquired resistance is one critical challenge to ALK-targeted therapies, and thus many researchers are exploring methods to overcome drug resistance during ALK-targeted therapies. Developing new generations of ALK–TKIs and novel targeting strategies with optimization (such as drug combinations) are two critical approaches to defending against ALK–TKI drug resistance.

Several years ago, crizotinib was established as the standard first-line therapy for advanced ALK-positive NSCLC, because it was demonstrated to be superior to standard chemotherapy (both progression-free survival and objective response rates) in patients with ALK-positive NSCLC [127,128,129,130]. However, crizotinib is far from perfect as a first-line therapy for advanced ALK-positive NSCLC, because of its poor penetration of the central nervous system and the inevitable development of crizotinib resistance during therapy. A newer generation of ALK-TKIs, such as ceritinib and alectinib, have demonstrated efficacy in the treatment of crizotinib-resistant ALK-positive NSCLC [112,121,126,131,132,133,134]. Also, researchers are investigating, in clinical studies, the potential of newer generations of ALK-TKIs as first-line therapies of advanced ALK-positive NSCLC [135,136,137,138]. Several second-generation ALK-TKIs may become first-line therapies of this cancer in future.

Investigators can compare candidate ALK-TKIs with chemotherapy in clinical studies to evaluate whether a particular ALK-TKI can be developed as a first-line therapy. Ceritinib is one of the most widely investigated ALK-TKIs that has the potential to be used as a first-line therapy [137,139,140]. One recent phase 3 clinical trial compared ceritinib with chemotherapy in previously untreated ALK-rearranged NSCLC [137]. This open-labeled, randomized study in untreated patients with stage IIIB/IV ALK-positive non-squamous NSCLC showed that ceritinib displayed superiority, over platinum-based chemotherapy, as the first-line treatment in these patients [137]. The median progression-free survival for patients in the ceritinib group was 16.6 months (95% CI 12.6–27.2) compared to 8.1 months (5.8–11.1) in the chemotherapy group (hazard ratio 0.55 (95% CI 0.42–0.73); *p* < 0.00001) [137]. Additionally, the overall intracranial response rates of the ceritinib group were higher compared to the chemotherapy group.

Furthermore, researchers are investigating whether other ALK-TKIs can become the first-line therapy for ALK-positive NSCLC, by comparing other ALK-TKIs with crizotinib in clinical studies [135,136,138]. Phase 3 clinical studies, with a head-to-head comparison of alectinib and crizotinib, have suggested that alectinib has the potential to become the first-line treatment of ALK-positive NSCLC [135,136]. In these studies, alectinib showed superior efficacy and lower toxicity compared to crizotinib in the primary treatment of patients with ALK-positive NSCLC [135,136]. In a recent randomized, open-label, phase 3 clinical study, researchers compared the efficacy and safety of brigatinib with those of crizotinib, in patients with advanced ALK-positive NSCLC who had not previously received ALK inhibitor treatment [138]. In this study, brigatinib, as compared to crizotinib, displayed superior efficacy against this cancer. The rate of progression-free survival was significantly higher that among patients in the brigatinib group than among those in the crizotinib group [138].

## 11. Future Research of ALK

Several vital questions in the research area of ALK include, but are not limited to: (1) What is the biological role of human ALK in embryonic development? (2) What is the biological role of extracellular cleavage and intracellular cleavage of ALK? (3) What is the biological role of ALK^ATI^ isoforms? (4) What is the biological role of ALK as a dependence receptor? (5) What is the 3-D structure of whole ALK or ALK ECD? And (6) how does drug resistance (such as ALK acquired resistant mutations) become established with the stress of ALK tyrosine kinase inhibition? Investigations related to these questions can help us to further understand the biological role of ALK. Moreover, this knowledge can also provide vital information to improve the strategy of ALK-targeted therapy. For instance, this information may help to develop novel drug combination strategies.

## 12. Summary

ALK is an RTK with many characteristics to explore in the future. Different aspects of ALK biology are summarized in this review. Because aberrant forms of ALK are related to multiple cancers, understanding different aspects of this protein provides essential information for us to understand the role of ALK in diseases. This knowledge is also crucial for exploring novel ALK-related therapies.

## Figures and Tables

**Table 1:** Summary of several novel ALK fusion proteins discovered recently.

Disease	Fusion Protein	Original Locus of Fusion Partner	References
Lung adenocarcinoma	VIT–ALK	2p22.2	[84]
Lung adenocarcinoma	GCC2–ALK	2q12.3	[85]
Melanocytic myxoid spindle cell tumor	FBXO28–ALK	1q42.11	[86]
Melanocytic myxoid spindle cell tumor	NPAS2–ALK	2q11.2	[86]
Spitz tumor	MLPH–ALK	2q37.3	[87]
Non-small-cell lung cancer	CUX1–ALK	7q22.1	[88]
Non-small-cell lung cancer	BCL11A–ALK	2p16.1	[89]
Non-small-cell lung cancer	STRN–ALK	2p22.2	[90]
Non-small-cell lung cancer	CMTR1–ALK	6p21.2	[91]
Inflammatory myofibroblastic tumor	A2M–ALK	12p13.31	[92]
Inflammatory myofibroblastic tumor	HNRNPA1–ALK	12q13.13	[93]
Inflammatory myofibroblastic tumor	IGFBP5–ALK	2q35	[94]
Inflammatory myofibroblastic tumor	THBS1–ALK	15q14	[94]
Inflammatory myofibroblastic tumor	NUMA1–ALK	11q13.4	[95]
Colorectal cancer	CAD–ALK	2p23.3	[96,97]
Glioma	PPP1CB–ALK	2p23.2	[98]
Gastrointestinal leiomyomas	FN1–ALK	2q35	[99]
Renal cell carcinomas	HOOK1–ALK	1p32.1	[100]
Renal cell carcinomas	STRN–ALK	2p22.2	[101]
Epithelioid fibrous histiocytoma	PRKAR2A–ALK	3p21.31	[102]
Epithelioid fibrous histiocytoma	MLPH–ALK	2q37.3	[102]
Endometrial cancer	EML4–ALK	2p21	[103]
Large B-cell lymphoma	GORASP2–ALK	2q31.1	[104]

**Table 2:** Summary information of several ALK inhibitors already used in clinical application.

ALK Tyrosine Kinase Inhibitors	Generation	Other Targets	Indicated Application	Some Mutations in ALK Kinase Domain with Resistance	References
Crizotinib	First generation	ROS1, MET, et al.	ALK+ or ROS+ metastatic non–small cell lung cancer (NSCLC)	EML4-ALK: L1196M; G1269A; G1202R; I1151T	[112,113,114,115,116,117,120,122]
Ceritinib	Second generation	ROS1, IGF-1R, InsR	ALK+ metastatic NSCLC after the failure of prior crizotinib therapy	EML4-ALK: G1202R; C1156Y; F1174C	[112,116,119,122]
Alectinib	Second generation	LTK, GAK	ALK+ metastatic NSCLC after the failure of prior crizotinib therapy	EML4-ALK: G1202R I1171T; V1180L	[118,119,121,122]
Brigatinib	Second generation	ROS1, EGFR	ALK+ metastatic NSCLC after the failure of prior crizotinib therapy	EML4-ALK: G1202R	[108,122,123,124,125,126]
